# Effect of different periodization models of combined training on the perception of barriers to physical activity in adults with obesity: A randomized clinical trial

**DOI:** 10.1002/ejsc.12030

**Published:** 2024-01-30

**Authors:** Fernanda Rosa, Willen Remon Tozetto, Jucemar Benedet, Giovani Firpo Del Duca

**Affiliations:** ^1^ Department of Physical Education Federal University of Santa Catarina Florianópolis Santa Catarina Brazil

**Keywords:** built environment, chronic disease, physical exercise, psychosocial impact

## Abstract

This study aimed to analyze the effect of combined training with linear and non‐periodized periodization and a control group on the perception of barriers to physical activity in adults with obesity. A randomized, controlled, and blinded clinical trial was conducted, comprising the control (CG), non‐periodized (NG), and linear periodization (PG) groups. Combined training was prescribed for 16 weeks. The NG kept the relative training intensity fixed (50%–59% of the heart rate reserve [HRres] and 10 to 12 repetition maximums [RMs]), while the PG linearly progressed (40%–49% HRres to 60%–69% HRres and 12 to 14 RMs to 8 to 10 RMs). The 19 barriers were obtained through a questionnaire. Generalized estimating equations (GEE) were used, adopting *p* ≤ 0.05 as a significant result, and individual responsiveness analyses. The CG significantly increased the score of lack of space (*Δ* = 0.6) and lack of equipment (*Δ* = 1.1) barriers. The NG (*Δ* = −0.4 and −0.6, respectively) and PG (*Δ* = −0.1 and −0.2, respectively) reduced their scores, showing a group*time interaction (*p* = 0.019 and 0.011, respectively). Through the individual responses, we verified a reduction in the number of barriers, notably in the groups with combined training (CG: 4 of 14; NG: 12 of 18; and PG: 10 of 17 individuals). Regardless of the form of periodization, the training groups reduced the score of some analyzed barriers. From a clinical point of view, individuals submitted to different forms of combined training periodization showed more expressive reductions in their total scores, when compared to the CG.

## Highlights

- The organization of training variables through periodization does not seem to significantly impact perceptions of barriers to physical activity.
- The combined training, regardless of periodization, was effective in reducing the perception of barriers to physical activity in adults with obesity.
- The reduction of obstacles to the practice of physical activity goes beyond the intrinsic motivations of the subjects, passing through the available infrastructure and its organization.

## 1 INTRODUCTION

Barriers to the practice of physical activity are factors that hinder or prevent adherence to the practice and can be classified as intrapersonal, interpersonal, and environmental (Grande & Silva, 2014). The perception of barriers can have a significant impact on the health of the entire population, especially those with chronic comorbidities (Fonseca et al., 2021). Among adults with obesity, barriers probably increase the risk of physical inactivity, and events such as falls, stigma, and embarrassment can predict this behavior (Hamer et al., 2021). In addition, the lack of time, motivation, financial resources, energy, poor physical preparation, and family and work demands are barriers to physical activity often reported by adults with obesity (Baillot et al., 2021; Call et al., 2019; Mcintosh et al., 2016; Rech et al., 2016; Stankevitz et al., 2017) and are related to sociodemographic and behavioral indicators, such as gender, education, and sedentary behavior (Llamas et al., 2020; Salmon et al., 2003).

Reducing barriers to the practice of physical activity is an important goal, and there is evidence that participation in an intervention with physical exercise can change the perception of barriers of individuals with obesity (Call et al., 2019). Thus, it is important to identify characteristics of physical training that are effective for greater participant engagement. One of the characteristics is the reduction of individuals' self‐regulatory resources, minimizing distractions for the practice, which originates from the projections and guidelines provided by the programs (Butryn et al., 2017). With this, people can focus on maintaining behavior in the face of barriers (decreased pleasure, muscle pain management, long‐term goals, and time). Another factor is the demystification of the practice of exercises, which, once performed, changes the perception of self‐efficacy (even if introjected initially) (Prochaska & Marcus, 1994). That is, individuals reassess their position in relation to exercise and the environment, perceiving themselves as capable of performing new functions and incorporating these into their daily lives.

In addition to this process, the extrinsic relations of the environment (when they occur) favor the maintenance of the behavior. As proposed by the theory of self‐determination, there is a continuum to achieve the maintenance of a new behavior, which is mostly initiated through an extrinsic motivational process (Ryan & Deci, 2008). Thus, barriers such as lack of company, lack of incentive, lack of adequate climate, and unsafe environment can be positively impacted by established social relationships. However, the guidelines given by the programs can reduce the autonomy of the practitioners, which is a long‐term problem, since the interruption of the programs acts directly on motivation (Ryan & Deci, 2008).

Regarding the type, combined training (aerobic and strength training performed in the same session) has been standing out against the isolated modalities in terms of adherence, as well as helping to improve anthropometric and metabolic parameters and being the main recommendation for physical activity to reduce of the weight (Schwingshackl et al., 2013). In addition to the type, periodization of training, a term which refers to the macro management (e.g., the macro, meso, and microcycle) of the process in relation to duration (time), enables the professional to anticipate and designate time intervals for the development and execution of specific physical fitness characteristics (Cunanan et al., 2018). However, it is little explored in relation to the perception of barriers, mostly in special populations.

Interventions based on the transtheoretical model of behavior change have been shown to be effective in modifying the level of physical activity (Freitas et al., 2020; Hepdurgun et al., 2020). However, they have methodological problems in their use (Mastellos et al., 2014; Tuah et al., 2011), which generate biases on the generalization of the results. The most common problems of these interventions are the lack of clarity about all methodological processes such as randomization, blinding, and detailing of the experiment prescription (Tuah et al., 2011), bringing difficulties in the inference about the barriers to the activity.

In any case, interventions based on the theory have been shown to be effective in reducing behaviors that are harmful to health, but their effectiveness in producing weight loss in obesity is unclear (Almeida et al., 2010; Freitas et al., 2020; Mastellos et al., 2014; Tuah et al., 2011). In fact, we did not find interventions based on physical exercise (even less related to periodization) focused on modifying the perception of barriers to physical activity. On the other hand, it is widely used in the competitive sports domain, in which there is evidence of the relationship between periodization and improvement in health outcomes in clinical populations (Strohacker et al., 2015).

The linear periodization model is characterized by a progressive increase in relative intensity with an equivalent decrease in volume (ACSM, 2009). On the other hand, non‐periodized training, characterized by the change in absolute intensity without manipulation of relative intensity (ACSM, 2009), has positive effects on physical fitness outcomes (Pinto et al., 2021), especially on the cardiorespiratory capacity and body composition (Clark et al., 2019). In general, nothing is known about the impact of the types of periodization of physical exercise on the perception of barriers to the practice of physical activities. However, we can formulate hypotheses based on experiences purportedly generated by each model of periodization.

The linear progression offers gradual progression, which could improve feelings of competence and self‐efficacy, reflecting on the reduction of the perception of barriers to physical activity. The hypothesis is that this procedure will impact more barriers linked to physical limitations and lack of interest since competence and self‐efficacy increase participants' motivation. The concern with appearance may be more impacted in this group.

Non‐periodized periodization starts at moderate intensities, which can lead to greater discomfort for beginners with obesity, such as joint pain and excessive fatigue (Marandi et al., 2013) and lack of motivation throughout the process because it does not bring changes in relative intensity, only absolute, and therefore less dynamic. However, the lower perception of alteration also provides a lower sensation of pain (Wasser et al., 2017). This is due to the continuity of the relative intensity, and therefore, the change in absolute intensity is continuous and gradual, while linear training tends to abrupt changes in its intensity. Furthermore, over time, the perception of fatigue and initial discomfort of non‐periodization is replaced, increasing the feeling of vigor and well‐being after training (Carraça et al., 2021).

Studies analyzing the effects of periodization of physical training in subjects with obesity usually present outcomes related to physical fitness, such as strength, cardiorespiratory capacity, and weight loss (Afonso et al., 2019; Said et al., 2021), and investigations into psychosocial outcomes, such as the perception of barriers to physical activity, are scarce in the literature. Thus, there is a gap in relation to studies that assess and compare perceptions of barriers from different periodization models. Given the above, the aim of this study was to analyze the effect of training combined with linear and non‐periodized periodization and a control group on the perception of barriers to physical activity in adults with obesity. The hypothesis of this work is that the practice of periodized physical exercises can promote a change in behavior and motivation in this population resulting in reduced barriers to physical activity (Call et al., 2019). Furthermore, our hypothesis is that the linear model will have a greater impact on the barriers associated with physical limitations, lack of interest, and concern with appearance, while the non‐periodized model will have a greater impact on barriers of pain perception, bad mood, fear of injury, feeling of pain, and lack of energy.

Maintaining the practice of exercises is an active process that requires strategies and techniques to maintain adherence, specifically in this population (Call et al., 2019). In this sense, to achieve this continuity, it is necessary to be self‐determined, that is, to reach the level of maintenance of the practice through autonomy, competence, and connection (Prochaska & Marcus, 1994), which is associated with achieving intrinsic motivation (Ryan & Deci, 2008). However, the investigated population tends to be in the opposite stages, that is, with no intention of acquiring the new behavior (pre‐contemplation) and being unmotivated. These factors are important to understand the adherence in the practice of physical activities. The assumption is that offering structured training can modify the behaviors of participants due to the reduction of barriers.

## 2 METHODS

### 2.1 Overview

The present study is a secondary analysis of a randomized, controlled, and blinded clinical trial, conducted from March to October 2018, with the aim of comparing the effect of different periodization models of combined training on outcomes of health markers and fitness in obese adults (Streb et al., 2019). The training period took place from May to August of the same year, being the months with the lowest temperatures of the year. Such information can be observed in the following link: (https://pt.weatherspark.com/h/y/30020/2018/Condi%C3%A7%C3%B5es‐meteorol%C3%B3gicas‐hist%C3%B3ricas‐durante‐2018‐em‐Florian%C3%B3polis‐Brasil#Figures‐ColorTemperature).

### 2.2 Participants

Participants were individuals of both sexes, aged between 20 and 50 years, and with obesity diagnosed via the body mass index (BMI) ≥30 kg/m^2^. The sample was intentional and non‐probabilistic. Information on the sampling procedure and methodological details of the research are available in the protocol study (Streb et al., 2019).

### 2.3 Eligibility criteria

‐ Not being involved in physical exercise programs (with a weekly frequency of more than 2 days) in the last 3 months;
‐ Not being a smoker (or who stopped smoking more than 6 months ago);
‐ Do not consume alcoholic beverages in excess (≥14 drinks in a week for men and ≥7 drinks for women);
‐ Not have osteomioarticular disease that limits the practice of physical exercise;
‐ Not using medication to control and/or treat obesity;
‐ Not having undergone a surgical procedure to reduce weight.

### 2.4 Randomization

Subjects were randomly allocated to one of the three experimental groups: control group (CG), non‐periodized group (NG), and linear periodization group (PG). The randomization process was performed on an *online* platform (www.randomizer.org) by researchers not involved in the intervention, and stratified by sex, age, and BMI, with a 1:1:1 ratio. The allocation list was hidden from the study evaluators. The same list was released to the coaches during the beginning of the intervention, and coaches did not participate in the evaluation phases, maintaining the evaluators' blinding.

### 2.5 Experimental procedure

The intervention was supervised by physical education professionals at all stages. The training groups underwent three weekly sessions of combined training with an average duration of 60 min, for a period of 16 weeks. Aerobic training, performed on an athletics track, consisted of continuous walking and/or running. The strength training, performed in the weight room, consisting of six exercises were performed involving large muscle groups: bench press, leg press, pull down, machine cross, squat on a leash, and low row. In order to familiarize the participants with the prescribed exercises, in the first week, the sessions lasted 30 min, with 15 min of low‐intensity aerobic exercise (30%–39% HRres) and 15 min of strength training, and with one series of 10 to 15 repetition maximums (RMs) of each exercise proposed in the intervention. After the familiarization week, the intervention sessions lasted an average of 1 hour, with 5 minutes of warm‐up, 30 min of aerobic training, 20 min of strength training, and a final 5 minutes of stretching. Two sets of each strength exercise were performed, with 60 s of rest between each set and exercise.

The two training groups performed two different types of periodization present in the literature, non‐periodized periodization, where there is no variation in intensity and relative volume, only absolute, and linear periodization, which presents a progressive decrease in volume with a concomitant increase in relative and absolute intensity (Minozzo et al., 2008).

The PG had a training model with increasing linear periodization, composed of three mesocycles of 5 weeks each. In the first mesocycle, the intensity of 40%–49% HRres was adopted for aerobic training, and for strength training, two sets of 12 to 14 RMs. In the second mesocycle, the intensity of the aerobic activity was between 50% and 59% HRres, and the strength training was performed with 10 to 12 RMs. In the third mesocycle, the participants reached the intensity of 60%–69% HRres, performing 8 to 10 RMs in strength training. The NG maintained aerobic training at moderate intensity (50%–59% HRres) and strength training with two sets of 10 to 12 RMs throughout the intervention period. At the end of each mesocycle, the resting heart rate was reassessed to adjust the target heart rate prescription in NG and PG. Both groups were instructed to increase the weight of the strength exercises when the maximum repetitions exceeded the target repetition range, in order to maintain the progressive load stimulus. After each mesocycle, the number of repetitions of strength exercises was changed only for the PG. The CG participants did not receive any intervention and were instructed to maintain their routine activities.

### 2.6 Assessments for sample characterization and exercise prescription

Data on sociodemographic variables were collected through an online questionnaire, involving gender (male and female), marital status (with and without a partner), skin color (white and others), employment status (employed and unemployed), age (years), and education (years of study). These questionnaires were given to all participants 4 weeks before the start of the program and reassessed 4 weeks after the last week.

To prescribe aerobic training by HRres, maximum and resting heart rates were used to calculate the ideal training zone, obtained by a portable heart rate meter (Polar S810i). The maximum heart rate was obtained by an incremental test (Libardi et al., 2011) during the initial estimatives of the study. To measure the resting heart rate, the participant was lying down with the heart rate monitor positioned on the thoracic region, and three one‐minute notes were taken, with a one‐minute interval between them. The reference value used was the average of the three observed measures. Only the resting heart rate was to be reevaluated at weeks 5 and 10, to adjust for possible changes in the HRR during the interventions.

All participants were tested on their one maximum repetition (RM) in bench press and leg press, in at least three different sessions, with intervals of 48 h between sessions, according to the guidelines proposed by Clark (1973). The increase in load (kilograms) will be indicated whenever participants perform the predicted series in the upper repetition range. More information and details can be found in the protocol study (Streb et al., 2019).

### 2.7 Outcomes assessment

To measure the perception of barriers to the practice of physical activity, the instrument used was the Perception of Barriers to the Practice of Physical Activities questionnaire, elaborated and validated in terms of reproducibility, applicability, and clarity by Martins and Petroski (2000). The instrument has the statement “Considering the factors listed below, indicate how often they represent, for you, factors that hinder your practice of physical activity”, and is composed of a list of 19 barriers evaluated on an ordinal scale (“always,” “almost always,” “sometimes,” “rarely,” and “never,” scored at 4, 3, 2, 1, and 0, respectively) (Martins & Petroski, 2000), with a general range from 0 to 76 points. Also, barriers were classified as intrapersonal (household chores, bad mood, fear of injury, physical limitations, mild pain and/or discomfort, lack of energy, lack of physical skills and financial resources, concern with appearance, lack of interest, and lack of knowledge), interpersonal (long working hours, family commitments, lack of company, and lack of encouragement), and environmental (lack of adequate climate, lack of available space, lack of available equipment, and unsafe environment). Intrapersonal barriers ranged from 0 to 44, and interpersonal and environmental barriers ranged from 0 to 16 points each. Lower perception of barriers was represented by lower scores, and greater perception by higher scores.

### 2.8 Data analysis

For the descriptive analysis of the continuous variables, values of mean, standard deviation, median, and interquartile range were used, and for categorical variables, absolute and relative frequencies. The normality of continuous data was assessed by viewing histograms and by the Shapiro–Wilk test. For the comparison of baseline groups, one‐way analysis of variance (ANOVA *one way*) with Bonferroni post hoc and the nonparametric Kruskal–Wallis equivalent was performed.

The effect of the intervention on barriers was verified using generalized estimating equations (GEE), expressed as mean and standard error, considering statistical significance as *p* ≤ 0.05. In significant cases, Bonferroni post hoc was adopted for multiple comparisons. The analyses were conducted by the protocol (PP), which considers the data of the participants who remained until the end of the intervention and have pre and post values in the barriers variables. Data were analyzed using IBM SPSS software version 21.0 (IBM Corp.). The magnitude of the difference (post–pre) between assessments was expressed as the mean difference (*Δ*). To calculate the magnitude of the intervention effect within each group, Cohen's *d* effect size was used. The effect sizes were considered as small (0.20 ≤ *d* < 0.50), medium (0.50 ≤ *d* < 0.80), and large (*d* ≥ 0.80) (Cohen, 1988). The GraphPad PRISM 7 was used to illustrate the overall score of barriers for each participant in the pre‐ and post‐intervention moments.

### 2.9 Ethical aspects

The study was approved by the Ethics and Research Committee on Human Beings (add protocol number) and registered in the Registry of Clinical Trials (add registration number).

## 3 RESULTS

**FIGURE 1:**
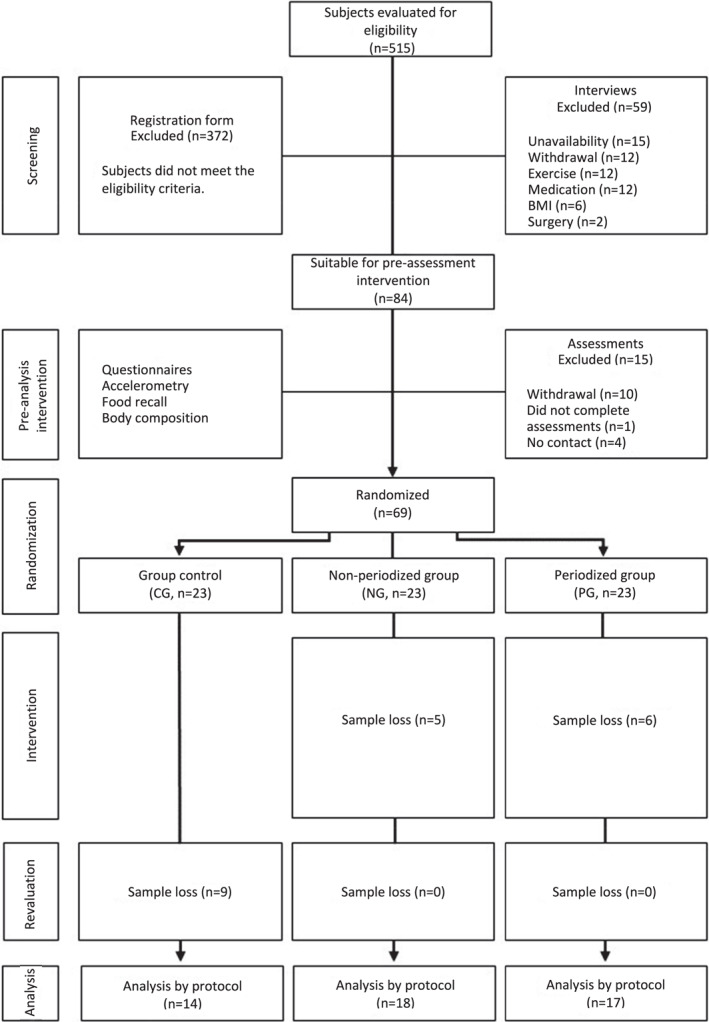
Flowchart of participants throughout the study.

Of the 515 subjects recruited for the study, 372 did not meet the eligibility criteria. During the interviews, 59 subjects were excluded, and in the period of pre‐intervention assessments, the sample loss was 15 individuals. Thus, 69 individuals initially participated in the study, aged between 20 and 50 years, 60.9% of whom were women. Participants were randomized into three groups stratified by sex, age, and BMI: CG (23), NG (23), and PG (23). During the intervention, there was a loss of samples, totaling 49 participants at the end of the study, distributed in CG (14), NG (18), and PG (17). Figure 1 summarizes the participation of individuals during the research.

Participants had a mean age and education of 34.7 (±7.2) and 15.9 (±2.8) years, respectively. Table 1 presents the comparison of groups from baseline sociodemographic variables.

**TABLE 1:** Characteristics of study participants (*n* = 69).

Variable	Pre‐intervention			*p*‐value
	CG (*n* = 23)	NG (*n* = 23)	PG (*n* = 23)	
	*n* (%)	*n* (%)	*n* (%)	
Sex				1.000
Male	9 (13.0)	9 (13.0)	9 (13.0)	
Female	14 (20.3)	14 (20.3)	14 (20.3)	
Current marital status				0.442
With a partner (a)	17 (24.7)	14 (20.3)	13 (18.8)	
No partner (a)	6 (8.7)	9 (13.0)	10 (14.5)	
Skin color				0.910
White	19 (27.6)	19 (27.6)	18 (26.0)	
Non‐white	4 (5.8)	4 (5.8)	5 (7.2)	
Current employment status				0.652^a^
Employed	20 (29.0)	17 (24.7)	18 (26.0)	
Unemployed	3 (4.4)	6 (8.7)	5 (7.2)	

		x̅ (±sd)		
Age (years)	34.1 (±7.6)	34.2 (±6.7)	35.6 (±7.4)	0.740
Education (years of study)	15.3 (±2.3)	16.0 (±3.1)	16.3 (±2.8)	0.332^b^
Body mass index (kg/m^2^)	33.2 (±2.4)	33.7 (±3.0)	33.6 (±3.2)	0.938^b^

Abbreviations: %, relative frequency; CG, control group; n, absolute frequency; NG, non‐periodized group; PG, periodized group; sd, standard deviation; x̅, mean.

^a^ Fisher's exact test.

^b^ Kruskal–Wallis test.

Per‐protocol analysis (Table 2) was used to assess the effect of the intervention. Significant group changes were observed in the post‐time period, where the barrier lack of adequate climate showed a difference between PG and NG, lack of company showed a difference between PG and NG and between PG and CG, and lack of energy between PG and CG. In terms of time, the changes were significant in the perceptions of physical limitations and pain/discomfort, which increased for participants in all groups at the end of the intervention. The interaction was significant just in time for the barriers of lack of available space and lack of available equipment, in which the CG increased the perception of pre to post intervention. Regarding the lack of the available space barrier, a difference was found in the pre values between NG and CG, with no difference in the post values. The barrier lack of available equipment showed a difference between PG and CG, and between NG and CG, both in the post‐time period, where the training groups had a lower perception of the barrier compared to the CG. In both barriers, there was an increase in perception by the CG, with a large magnitude of effect (Cohen's *d* = 0.83 and 1.11, respectively), while in the NG and PG they were reduced, observing a greater reduction for NG (NG *Δ* = −0.4; PG *Δ* = −0.1 and NG *Δ* = −0.6; G = PG *Δ* = −0.2, respectively), with a small magnitude of effect (Cohen's *d* = 0.44 and 0.48, respectively). There were no significant differences in the general scores of the barriers.

**TABLE 2:** Analysis of the effect of the intervention on the perception of barriers to the practice of physical activity.

	Pre‐intervention	Post‐intervention	*Δ*	Cohen's d	*p*‐value		
	*X* (±se)	*X* (±se)			*g*	*t*	g*t
By protocol (*n* = 49)							
Overall score							
CG	26.2 (±2.5)	30.6 (±2.1)	4.4	0.53			
NG	30.3 (±2.0)	26.3 (±1.9)	−4.0	0.51	0.154	0.685	0.204
PG	27.0 (±2.2)	24.2 (±2.0)	−2.8	0.33			
Long working hours							
CG	2.2 (±0.3)	2.6 (±0.2)	0.4	0.39			
NG	1.6 (±0.3)	2.3 (±0.3)	0.7	0.67	0.051	0.278	0.115
PG	2.7 (±0.3)	2.4 (±0.2)	−0.3	0.35			
Family commitments							
CG	1.9 (±0.4)	1.4 (±0.2)	−0.5	0.43			
NG	1.3 (±0.3)	1.6 (±0.2)	0.3	0.30	0.690	0.158	0.081
PG	1.8 (±0.2)	1.2 (±0.2)	−0.6	0.66			
Lack of proper climate							
CG	1.3 (±0.3)	1.5 (±0.2)	0.2	0.23			
NG	1.5 (±0.2)	1.7 (±0.2)	0.2	0.21	0.013	0.191	0.860
PG	0.9 (±0.2)	1.3 (±0.2)^a^	0.4	0.49			
Lack of available space							
CG	0.8 (±0.2)	1.4 (±0.2)^b^	0.6	0.83			
NG	1.7 (±0.3)^c^	1.3 (±0.2)	−0.4	0.44	0.267	0.968	0.019
PG	1.2 (±0.3)	1.1 (±0.2)	−0.1	0.18			
Lack of available equipment							
CG	1.0 (±0.4)	2.1 (±0.2)^b^	1.1	1.11			
NG	1.8 (±0.3)	1.2 (±0.3)^c^	−0.6	0.48	0.593	0.564	0.011
PG	1.4 (±0.3)	1.2 (±0.2)^c^	−0.2	0.16			
Domestic chores							
CG	1.7 (±0.3)	1.9 (±0.2)	0.2	0.23			
NG	1.7 (±0.2)	1.1 (±0.2)	−0.6	0.61	0.198	0.612	0.169
PG	1.4 (±0.3)	1.4 (±0.2)	0	0.06			
Lack of company							
CG	2.0 (±0.3)	2.2 (±0.3)	0.2	0.22			
NG	2.6 (±0.2)	1.8 (±0.3)	−0.8	0.69	0.045	0.144	0.267
PG	1.8 (±0.4)	1.2 (±0.2)^a^ ^,^ ^d^	−0.6	0.45			
Lack of encouragement from family and/or friends							
CG	1.4 (±0.3)	1.6 (±0.2)	0.2	0.23			
NG	1.8 (±0.3)	1.2 (±0.2)	−0.6	0.51	0.181	0.165	0.167
PG	1.4 (±0.3)	0.8 (±0.3)	−0.6	0.35			
Lack of financial resources							
CG	2.1 (±0.4)	2.3 (±0.3)	0.2	0.17			
NG	2.1 (±0.3)	1.6 (±0.3)	−0.5	0.51	0.475	0.708	0.397
PG	1.9 (±0.3)	2.0 (±0.2)	0.1	0.05			
Bad mood							
CG	1.1 (±0.2)	1.5 (±0.3)	0.4	0.48			
NG	1.3 (±0.2)	1.2 (±0.2)	−0.1	0.17	0.939	0.953	0.310
PG	1.4 (±0.2)	1.1 (±0.2)	−0.3	0.32			
Fear of being injured							
CG	0.9 (±0.2)	0.9 (±0.2)	0	0.09			
NG	0.6 (±0.2)	1.1 (±0.2)	0.5	0.53	0.829	0.429	0.475
PG	0.9 (±0.2)	1.0 (±0.3)	0.1	0.06			
Physical limitations							
CG	0.6 (±0.1)	0.8 (±0.3)	0.2	0.31			
NG	0.6 (±0.2)	1.2 (±0.2)	0.6	0.75	0.359	0.032	0.478
PG	0.9 (±0.2)	1.1 (±0.2)	0.2	0.21			
Pain and/or discomfort							
CG	0.6 (±0.2)	1.2 (±0.3)	0.6	0.67			
NG	0.8 (±0.2)	1.5 (±0.2)	0.7	0.80	0.432	0.016	0.291
PG	1.0 (±0.2)	1.1 (±0.2)	0.1	0.08			
Lack of energy							
CG	2.5 (±0.3)	2.6 (±0.2)	0.1	0.08			
NG	2.7 (±0.2)	2.2 (±0.2)	−0.5	0.52	0.021	0.108	0.372
PG	2.4 (±0.3)	1.7 (±0.3)^d^	−0.7	0.60			
Lack of physical skills							
CG	1.1 (±0.2)	1.1 (±0.3)	0	0.08			
NG	1.6 (±0.2)	1.0 (±0.2)	−0.6	0.69	0.553	0.166	0.457
PG	1.4 (±0.3)	1.2 (±0.3)	−0.2	0.21			
Lack of knowledge							
CG	1.0 (±0.2)	1.4 (±0.3)	0.4	0.39			
NG	1.8 (±0.3)	1.0 (±0.2)	−0.8	0.79	0.513	0.379	0.063
PG	1.2 (±0.3)	1.1 (±0.2)	−0.1	0.12			
Unsafe environment							
CG	1.1 (±0.3)	1.2 (±0.2)	0.1	0.08			
NG	1.4 (±0.3)	0.9 (±0.2)	−0.5	0.51	0.874	0.546	0.265
PG	1.0 (±0.2)	1.1 (±0.3)	0.1	0.12			
Concern about appearance							
CG	1.2 (±0.3)	1.3 (±0.3)	0.1	0.08			
NG	1.2 (±0.3)	1.0 (±0.3)	−0.2	0.20	0.545	0.606	0.844
PG	1.1 (±0.2)	0.9 (±0.2)	−0.2	0.20			
Lack of interest in practice							
CG	1.6 (±0.2)	1.7 (±0.3)	0.1	0.08			
NG	2.2 (±0.2)	1.5 (±0.3)	−0.7	0.72	0.117	0.428	0.139
PG	1.3 (±0.2)	1.5 (±0.3)	0.2	0.18			

*Note*: ±se, standard error; CG, control group; *g*, difference between the groups; g*t, interaction between the group and time; NG, non‐periodized group;PG, periodized group; *t*, difference between the times; *X*, mean; *Δ*, difference between post‐ and pre‐intervention.

^a^ Significant difference between *g* and NG.

^b^ Significant intragroup difference (pre vs. post).

^c^ Significant difference between g*t and CG.

^d^ Significant difference between *g* and CG.

**FIGURE 2:**
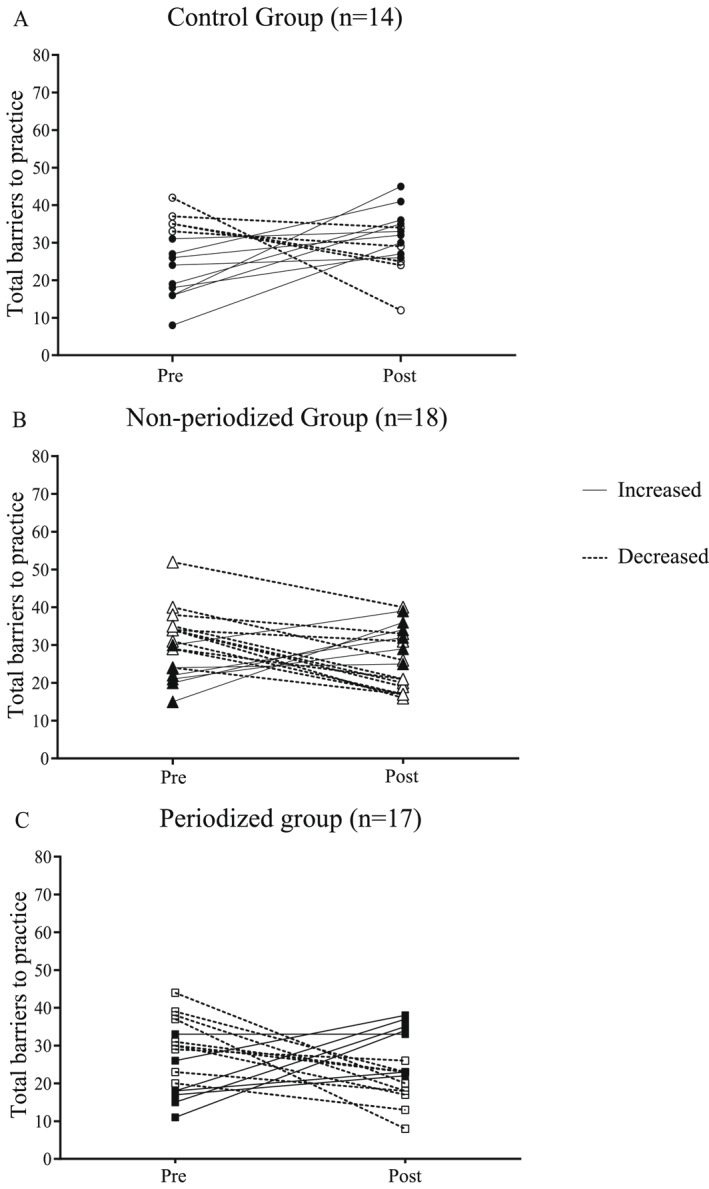
Individual scores of the total score of barriers to physical activity in the pre‐ and post‐intervention periods.

Figure 2 presents individual responses to perceived barriers to physical activity in each intervention participant, according to their allocation group. It was found that few subjects in the control group showed a reduction in the perception of barriers (4 out of 14, or 28.6%), while those belonging to the non‐periodized combined training groups and with linear periodization had a reduction in the total score of perceived barriers (12 out of 18 in non‐periodized and 10 out of 17 in linear periodized or 66.7% and 58.8% reduction of barriers, respectively).

## 4 DISCUSSION

This study aimed to analyze the effect of training combined with linear and non‐periodized periodization and a control group on the perception of barriers to physical activity in adults with obesity. From a clinical point of view, both training combined with linear and non‐periodized periodization can contribute to the reduction of barriers. On the other hand, in the control group, few people reduced the overall perception of barriers, while more than half of the people in the exercise groups reduced it. This reinforces the condition of physical exercise as an important facilitating agent for other physical activities of daily life.

Other studies that evaluated barriers to physical activity in the adult population with obesity after a period of physical exercise intervention also observed a reduction in barriers at the end of the intervention (Gallagher et al., 2006). After 12 months of aerobic exercise, Call et al. (2019) observed a reduction from 20 to 14 in the average score of total barriers, considering a scale from 0 to 63 points. In the study of Thomson et al. (2016), in a shorter intervention period (5 months), the average barrier score also reduced, from 30 to 25 in the group with aerobic training and from 28 to 26 in the group with combined exercise, considering a scale of 14 to 56 points. On the other hand, a qualitative study (Venditti et al., 2014) showed an increase in the perception of barriers at the end of 6 months of moderate physical activity. Unlike our findings, in which the only intervention proposed was the physical exercise program, these interventions consisted of multidisciplinary strategies for weight loss, encompassing behavioral education and/or diet, and not just exercise. Our results show that the reduction of barriers to physical activity can occur through simple exposure to physical exercise, regardless of interventions in other health‐related behaviors.

The control group that did not participate in the training showed an increase in the perception of barriers to physical activity over time. One hypothesis is that perception of barriers to the practice of physical activity is in constant change, as they are sensitive to events in the lives of the subjects and the environment. In this same context, one possibility is that the change in the perception of barriers after the period of the intervention is not directly related to it, but to some specific event in the individual's routine. However, there is a lack of evidence to support the hypothesis. We cannot rule out the possibility that the allocation of these subjects to the control group has generated demotivation, which over the 16 weeks has increased the perception of barriers. According to the theory of self‐determination, the stages are cyclical and bidirectional (Ryan & Deci, 2008) insofar as participation in a physical exercise program guided and structured by researchers must have provided extrinsic motivations through external and introjected regulations, but when announced as belonging to the control group and who should maintain the previous routine, it demotivates the participants who, as a result, have a greater perception of barriers at the end of the project.

There is evidence that interventions with physical exercise as part of their proposal, in which the result in relation to the perception of barriers was not uniform, modify barriers in positive and negative directions (Call et al., 2019). The lack of the time barrier, for example, can be seen differently by individuals after the intervention: While for some the exercise can provide greater motivation to organize time and consequently lower perception of this barrier, for others, the fact of being involved in one more demand can contribute to an increase in perception.

After 16 weeks of intervention, the perception of lack of available space and lack of equipment barriers increased in the CG and decreased in both exercise groups, which in a way was expected, considering the condition of offering a place and equipment to perform the aerobic and strength training. Often, the practice of physical activity does not depend on an individual decision only (Reichert, 2011), but is conditioned to the environment in which the individual is inserted. In addition to infrastructure, the availability of safe spaces can help adults to engage in exercise routines (Rech et al., 2016); after all, it is not expected that motivating a subject to change their behavior in a busy environment is effective (Sallis et al., 2012). In addition, sometimes obese individuals are afraid or ashamed to enter environments intended for physical activity, as well as due to fear of embarrassment and stigma (Hamer et al., 2021). Thus, the interaction of the study participants with their peers and the contact with the physical education professionals who guided the intervention may have provided an environment with good reception and a feeling of belonging, which may have helped to change the perception regarding the “space” for physical activity.

Although without significant group × time interaction, it is worth mentioning the direction that some barriers commonly faced by the study population presented after the intervention. The perception of barriers lack of company and lack of energy increased in the control group and was reduced in the training groups, suggesting a positive effect of combined training. The initially reported lack of company may have been compensated by the welcoming environment and interaction with peers. The reduction of the barrier of lack of energy in the training groups can also be related to the aforementioned environmental conditions (welcoming environment and interaction with peers), capable of impacting the subjects' motivation, and with the physiological benefits that combined exercise provides (Ryan & Deci, 2008; Schwingshackl et al., 2013), regardless of periodization. That is, when reporting a lack of energy, the individual may be referring to a physiological issue, or the will and motivation to practice. The perception of lack of adequate climate, physical limitations, and pain/discomfort increased for all groups. Changes in the climate affect the perception of this barrier, due to the preferences of the individuals, and perhaps at the end of the intervention, the climate was not as satisfactory as at the beginning. It should be noted that the intervention took place mostly in the winter period.

About the physical limitations and pain/discomfort barriers, these may be linked to a greater body of awareness at the end of the intervention, where subjects with obesity possibly came to realize previously unknown limitations due to the fact that they were not involved in an exercise routine. Such results indicate that the perception that pain is a barrier to the practice has increased. However, we cannot say that the pain sensation has increased. In fact, in that same sample, Tozetto et al. (2022) observed that the groups that trained reduced the sensation of pain after the intervention. Confirming this statement, Wasser et al. (2017) identified in their review that several training programs (including aerobic and resistance) are able to reduce body pain in this population after the training period. However, it should be mentioned that none of the studies included by Wasser et al. (2017) used combined training. Thus, the results point out that despite the combined training reducing body pain, the perception that pain is a limiting factor for the practice is increased, and that it is necessary to take into account the increase in the initial sensation of pain during the process for the behavior maintenance in this population. This becomes relevant since, with the sensation of increased pain, psychological aspects such as competence and, mainly, self‐efficacy, can be compromised, favoring an increase in the perception of the barrier of physical limitations (Prochaska & Marcus, 1994). Such findings need new evidence to be understood.

It is also necessary to observe the uniqueness of the sample recruited to understand these results since they did not have other health problems besides obesity, such as diabetes mellitus TII or hypertension (very common in this population), were not smokers or consumed too much alcoholic beverages, did not present musculoskeletal diseases or limitations, and did not use medication. Such limitations of the study make the studied sample to be considered a model with a smaller range of damages related to obesity in general and possibly not feeling the barriers related to pain in their daily lives. This can be reinforced by another limitation of the sample, that is, of not being involved with physical exercise programs in recent months.

It is necessary to adopt effective strategies to maintain this behavior in this population. An effective strategy found in the literature is early weight loss, which motivates the participant due to the rapid result (Burgess et al., 2017). However, many of these studies are based on bariatric surgeries, being a drastic and last resort strategy for the treatment of obesity (Kolotkin & Andersen, 2017). Even actions focused only on dietary and exercise changes can lead to frustration over time due to the difficulty of maintaining weight, as more aggressive strategies are needed to achieve rapid weight loss. Kushner and Foster (2000) point out that the primary objective of obesity interventions is to improve the patient's outlook and not just weight loss. Another determining factor is the practitioner's mood (Burgess et al., 2017). To keep this population motivated to practice, it is necessary to create a positive and supportive environment, in which the practitioner creates significant bonds (Burgess et al., 2017). This is related to the improvement in mental health, which was previously observed in our sample after the intervention (Tozetto et al., 2022) but which seems not to have influenced the lower perception of barriers.

It should be noted that the strategies for maintaining the behavior of people with obesity in exercise programs are still little explored in the literature (Almeida et al., 2010; Freitas et al., 2020) and were not part of the maintenance strategies of the investigated project. Even if insufficient, some strategies can be found in the literature. Hepdurgun et al. (2020) observed that active strategies are more effective for adherence to the behavior change program than passive strategies such as handing out a leaflet with guidelines. The same was reported by Freitas et al. (2020) in a behavior change program for people with obesity in the Brazilian public health system. The study compared two groups with the same physical activity and eating habits; however, one group carried out a health education program based on the transtheoretical model of behavior change. The program focused on a reflective, participatory, and proactive problem‐solving approach, in order to promote the autonomous practice of healthy habits and overcome barriers encountered (Freitas et al., 2020). In addition to significant weight reduction, a significant shift from pre‐action to the action stage has been reported.

In our study, we did not use strategies to act on the barriers and even so, a reduction, even without statistical significance, can be observed in the direction of the overall score of the intervention groups (and also by viewing the individual scores in Figure 2). This may be related to what the transtheoretical model itself addresses, since the subject is already inserted in the exercise routine (action) and can perceive the environment and the interactions that take place there, moving from the imaginary to the real (Prochaska & Marcus, 1994) adding this to motivational feedback from individuals present at the practice (even if unintentional), increasing extrinsic motivation among participants (Ryan & Deci, 2008). However, we cannot say that this reduction in barriers in the intervention groups was sufficient to maintain the behavior after the end of the program.

The effect of combined training (PG and NG), from the individual scores of the subjects (Figure 2), reinforces the importance of visualizing the results beyond their statistical significance. In randomized clinical trials, clinical relevance also needs to be considered, and, for that, one must take into account whether a given intervention will make a real difference in the subject's life, contemplating aspects such as the cost‐effectiveness of implementation and durability of effects (Fisher et al., 2018; Ranganathan et al., 2015). In this study, the reduction of barriers predominated in the training groups (62.8% in the training groups vs. 28.6% in the control group) and taking into account that the management of people with chronic diseases, such as obesity, requires long periods of treatment; these findings are relevant to clinical practice. As combined training is incorporated into the daily lives of subjects with obesity, important progress related to different outcomes is more effectively visualized; among these is the reduction of barriers to its practice. However, it is not possible to guarantee that individuals who practice physical exercises will present a reduction in the perception of barriers.

This is because individual results tend to have 2 times more variation than group results, as collective results represent an estimate of only 68% of the actual sample results (Fisher et al., 2018). Intragroup heterogeneity (or even sub‐samples) explains why different results occur under the same treatment, such as the complexity of human behavior (Fisher et al., 2018; Howard & Hoffman, 2018), and therefore, our collective findings cannot be translated as absolute truths for all individuals in the studied population. On the contrary, our findings point to the need to know the barriers to practice before starting training so that relationships between practitioners, training conduction, social relations, and objectives of participants and trainers can be established. Another possibility that cannot be ruled out is the possibility of a regression bias to the mean in the individual results since, in general, the initial values of the groups are inverted in the final values (i.e., smaller initial values have higher final values and vice versa). Regression bias to the mean occurs when more extreme values tend to approach the mean or the extreme value when reassessed (Barnett et al., 2005), which would result in a lack of validity of the questionnaire used for this context or a large variability for the perception of barriers.

We conducted this study knowing that physical exercise has positive effects on several psychosocial factors (Baillot et al., 2020). Our results indicate that the training periodization does not seem to interfere with statistical significance in perception of barriers. However, from a clinical perspective, the training groups reduced their perception of barriers. This denotes that greater focus is needed on creating a favorable environment for training maintenance for this population. This conclusion was observed for other variables such as health‐related quality of life and sleep in this sample (Leonel et al., 2022; Tozetto et al., 2022).

Unlike other studies that evaluated the perception of barriers from interventions in multiple behaviors (Call et al., 2019; Gallagher et al., 2006; Thomson et al., 2016), our study stands out for being an intervention exclusively with physical exercise, and, in addition, comparing different types of periodization. Furthermore, both exercise groups had intensity parameters constantly reassessed for prescription adjustments, and obtained the same total training volume, allowing an equivalent comparison. The study also has limitations, the main one being the sample size, which may have limited inferences. In addition, the fact that the subjects voluntarily participated in an exercise intervention reveals a predisposition to practice physical activity, contributing to a relatively low report of barriers at baseline and, consequently, a smaller amplitude for the intervention impact. Another limitation is related to the instrument used, which was designed for the adult population, but not specifically for the obese population.

The study did not observe differences between the forms of periodization, but combined training, regardless of periodization, was effective in reducing the perception of barriers to physical activity in adults with obesity. The perception of environmental barriers, such as lack of space and lack of equipment, were the ones that showed the greatest reduction, which allows us to highlight that the reduction of obstacles to the practice of physical activity which goes beyond the intrinsic motivations of the subjects, passing through the available infrastructure and its organization. Regarding the impact of periodization on the perception of barriers to the practice of physical activity, studies with a larger sample size, as well as with a qualitative approach, may be more effective to clarify this relationship.

## CONFLICT OF INTEREST STATEMENT

The authors report no conflict of interest.
